# Five years of the Institute on Methods and Protocols for Advancement of Clinical Trials in ADRD (IMPACT‐AD)

**DOI:** 10.1002/alz.70441

**Published:** 2025-07-14

**Authors:** Joshua D. Grill, Margaret D. Mastrolorenzo, Heather M. Snyder, Maria C. Carrillo, Ronald C. Petersen, Paul Aisen, Reisa Sperling, Kedir Hussen, Rema Raman

**Affiliations:** ^1^ Institute for Memory Impairments and Neurological Disorders Departments of Psychiatry and Human Behavior and Neurobiology and Behavior University of California, Irvine Irvine California USA; ^2^ Alzheimer's Therapeutic Research Institute University of Southern California San Diego California USA; ^3^ Division of Medical and Scientific Relations and Medical Affairs Alzheimer's Association Chicago Illinois USA; ^4^ Department of Neurology Mayo Clinic Rochester Minnesota USA; ^5^ Department of Neurology Massachusetts General Hospital, Harvard Medical School Boston Massachusetts USA

**Keywords:** clinical trials, education, training

## Abstract

**BACKGROUND:**

First held in 2020, the Institute on Methods and Protocols for Advancement of Clinical Trials in ADRD (IMPACT‐AD) is a program to train the next generation of Alzheimer's disease (AD) and related dementias (AD/ADRD) clinical trialists.

**METHODS:**

IMPACT‐AD includes didactic, workshop, and small group components.

**RESULTS:**

IMPACT‐AD has trained 18 alumni–scholars (accepted from 424 applicants), of whom 67% were female. Forty‐eight (26%) were the first in their family to attend college. Scholars included individuals from all racial and ethnic groups. Each year, participants demonstrated increased learning about AD/ADRD clinical trials through a pre/post‐test model of knowledge assessments. Among those completing annual follow‐up surveys, >84% remain in AD/ADRD trials careers, and 80% have experienced career advances or milestones. In the latter group, > 90% indicated that participating in IMPACT‐AD contributed to this career growth.

**DISCUSSION:**

IMPACT‐AD is a novel educational program that is achieving its goals.

**Highlights:**

- Over 5 years, the Institute on Methods and Protocols for Advancement of Clinical Trials in ADRD (IMPACT‐AD) has trained 188 scholars.
- The course has been accessible to a broad range of trainees who were diverse in professional and demographic backgrounds.
- Objective assessments indicate that participants gain knowledge through participation.
- Eighty‐four percent of trainees have remained in Alzheimer's disease and related dementias (ADRD) trials careers.
- Eighty percent of alumni have experienced career advances; nearly all indicated that participating in the course contributed to their success.

## 1 BACKGROUND

Evidence‐based medicine relies upon well‐designed and rigorously performed clinical trials to instruct appropriate care and improvements in that care. Through the course of medical and scientific education, minimal training is included on the design, conduct, or even interpretation of data from clinical trials. Thus, investigators to conduct these studies are few and often left to “learn on the job,” risking delays and other challenges in treatment development due to an inadequate workforce.

Alzheimer's disease (AD) and AD and related dementias (ADRD) are a public health crisis^1^, ^2^ for which federal law dictates the development of disease‐slowing and preventative therapies.^3^ A major challenge in addressing these crises is an inadequate supply of clinicians with the expertise to provide the needed care for an aging population.^4^, ^5^, ^6^, ^7^, ^8^ A similar, if not more extreme, issue exists in AD/ADRD research, in which there is a need to increase the number and supply of qualified investigators.^9^

As of 2024, a growing pipeline included >125 AD/ADRD candidate therapies in trials^10^ and even more trials of non‐pharmacologic and caregiver interventions.^11^ There is a need to ensure optimal testing of these treatments alone and in combination^12^ to identify their most effective uses. In addition to clinical expertise, AD/ADRD trial investigators must possess a diverse and increasing array of knowledge and skills, including understanding of evolving trial designs and research methods,^13^, ^14^ neuropsychopharmacology, and biostatistics.^15^ The complexity of AD/ADRD trials requires a team science approach. Physicians, biostatisticians, neuropsychologists, biomarker scientists, and ethicists all play critical roles in trial design. Trial conduct requires these specialties, as well as individuals who can carry out protocols and manage trial data, including coordinators, psychometricians, informaticians, and others. Moreover, to ensure that trials are inclusive and accessible to the greater disease‐suffering population, trial teams should be similar in terms of their demographics and lived experiences to the patients they aim to enroll.^16^, ^17^

In 2019, we established the Institute on Methods and Protocols for Advancement of Clinical Trials in ADRD (IMPACT‐AD)^18^ to address the low availability of skilled AD/ADRD trialists. In this report, we describe the first 5 years of conduct for IMPACT‐AD, including interest in the course, a description of the trainees who have participated, and the impact on trainees’ knowledge and career advances.

## 2 METHODS

### 2.1 Course structure

IMPACT‐AD is a multi‐day training course providing focused instruction on the design, conduct, and analysis of AD/ADRD clinical trials. The first 2 years of the course were held remotely due to the COVID pandemic. The subsequent 3 years have been held in person in San Diego, California, USA.

The course includes two tracks.^18^ A “fellowship track” is funded primarily by the National Institutes of Health and emphasizes current and future principal investigators. A “professional track” emphasizes key members of AD/ADRD trial teams and is primarily funded by the Alzheimer's Association. The fellowship track includes individuals interested in designing and leading their own trials (i.e., principal investigators). The professional track includes additional members of trial teams, such as study clinicians, psychometricians, coordinators, regulatory specialists, and other professionals.

The course includes short didactic lectures on AD/ADRD trial topics, as well as a variety of workshops and debates to provide opportunities for active learning.^19^, ^20^, ^21^ Didactic sessions include thematic organization, including: AD/ADRD trial designs; design and analysis; ADRD trial ethics; participant‐related issues; populations, indications, and outcomes; next generation outcomes; statistical considerations; and study management/serving as principal investigator.^18^ Active learning workshops are designed to enhance skills that will permit trainees to flourish in their careers as well as their science. This includes exercises on effective scientific communication, trial publications, sessions on study leadership, and evening activities in which established investigators share personal career stories and discuss work/life balance. Special emphasis is placed on ensuring adequate time for interaction between trainees and faculty and learning through discussion. The two tracks are conducted simultaneously, beginning with a welcome evening event that includes all trainees, faculty, and a keynote speaker. The professional track is then followed by 2.5 days of training, while the fellowship track extends an additional 2 days for a total of 4.5 days of training. These days emphasize the role of the principal investigator in AD/ADRD trials and include small working group sessions in which these trainees work closely with clinical and biostatistics faculty to develop an AD/ADRD trial protocol with a 1:1 trainee‐to‐faculty ratio.

### 2.2 Course faculty

Highly experienced AD/ADRD trialists serve as the faculty of IMPACT‐AD.^18^ This primarily includes site principal investigators and unit leaders from the Alzheimer's Clinical Trial Consortium (ACTC, www.actcinfo.org). Most faculty attend the duration of the course, providing extensive additional opportunities outside of structured course components for the trainees to receive vital mentorship and guidance.^22^, ^23^

### 2.3 Learning, satisfaction, and long‐term outcome assessments

At the course outset, knowledge of AD/ADRD trials is assessed through subjective and objective assessments. Each day of the course, trainees are asked to assess the value, quality, and impact of each course lecture, workshop, and other elements, including rating the quality of the presentation being delivered by the specific faculty member(s) for each course component. At the conclusion of the professional track and then the fellowship track, objective assessments are again performed to measure learning outcomes. These assessments, along with faculty feedback and annual meetings with a course steering committee, are considered each year by the course leadership to support iterative improvement.

Annual assessments of course alumni–scholars are conducted remotely to monitor career milestones, retention in the AD/ADRD space, and the frequency with which fellowship track trainees progress in the conduct of their trial protocol. In all cases, course alumni–scholars are asked to assess whether participation in the IMPACT‐AD program contributed to their career development.

## 3 RESULTS

### 3.1 Participants

**FIGURE 1 alz70441-fig-0001:**
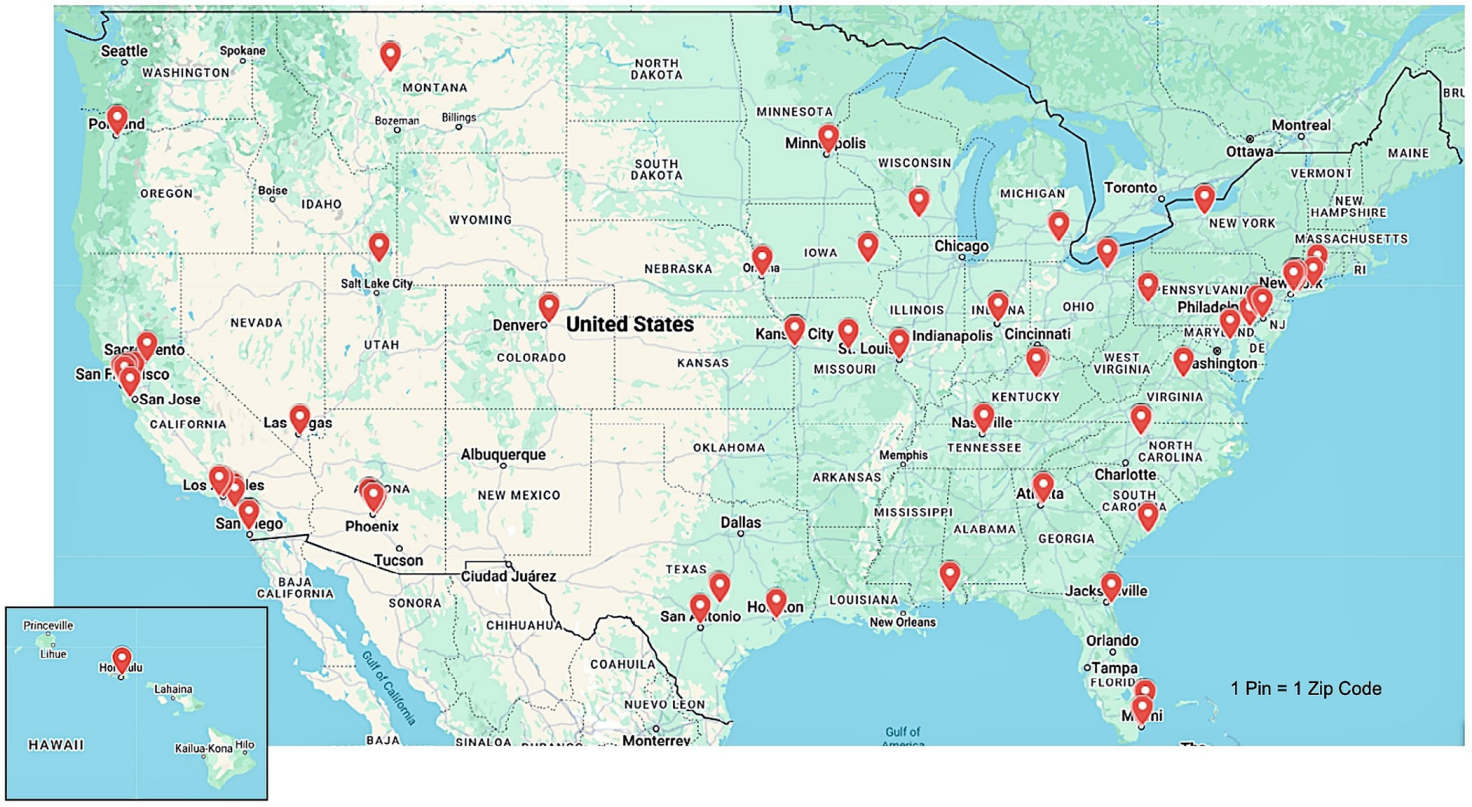
Distribution of IMPACT‐AD alumni–scholars across the United States. Each pin represents the institutional home of at least one IMPACT‐AD alumni–scholar. IMPACT‐AD, Institute on Methods and Protocols for Advancement of Clinical Trials in ADRD.

More than 420 unique individuals have applied to participate in IMPACT‐AD. Among these, 189 have been selected, 89 in the fellowship track and 100 in the professional track. This included 20 trainees in the professional track each year. The fellowship track expanded from 15 to 20 trainees in 2022. Table 1 describes the demographics of applying and accepted participants across the 5 years of the course. Across the tracks, 67% of trainees have been women, and 59% have self‐reported coming from a group underrepresented in science based on race, ethnicity, or sexual orientation (Table 1). Twenty‐nine (29%) trainees in the professional track and 19 (21%) in the fellowship track were the first in their families to attend college. Among fellowship track participants, a broad array of scientific backgrounds is represented, such as neurologists, psychiatrists, neuropsychologists, occupational therapists, and neuroscientists. Overall, nearly half of trainees have been from sites outside of the ACTC network. In fact, considering trainees’ institutional homes, 37 US states have been represented (Figure 1).

**TABLE 1:** IMPACT‐AD applicant and trainee demographics^a^.

	Professional track		Fellowship track	
Self‐reported characteristic	Applied (*n* = 225)	Selected (*n* = 100)	Applied (*n* = 199)	Selected (*n* = 89)
Sex				
Female	163 (72%)	65 (65%)	128 (64%)	61 (69%)
Male	62 (28%)	35 (35%)	71 (36%)	28 (32%)
Race				
African American or Black race	17 (8%)	12 (12%)	19 (10%)	9 (10%)
Alaska Native or American Indian race	2 (1%)	1 (1%)	0 (0%)	0 (0%)
Asian race	37 (17%)	15 (15%)	46 (23%)	17 (19%)
More than one race	12 (5%)	7 (7%)	9 (5%)	2 (2%)
Native Hawaiian or Pacific Islander race	1 (0.4%)	1 (1%)	0 (0%)	0 (0%)
Other race	8 (4%)	4 (4%)	7 (4%)	4 (5%)
Prefer not to answer/no response	9 (4%)	4 (4%)	4 (2%)	1 (1%)
White race	139 (62%)	56 (56%)	114 (57%)	56 (63%)
Ethnicity				
Hispanic or Latino ethnicity	30 (13%)	18 (18%)	19 (9%)	12 (14%)
Non‐Hispanic or Latino ethnicity	187 (84%)	79 (79%)	174 (87%)	76 (85%)
Prefer not to answer/no response	8 (4%)	3 (3%)	6 (3%)	1 (1%)
Sexual orientation				
Heterosexual or straight	179 (81%)	81 (81%)	71 (86%)	76 (85%)
LGBTQ+	22 (10%)	12 (12%)	12 (6%)	7 (8%)
Prefer not to answer/no response	24 (11%)	7 (7%)	16 (8%)	6 (7%)
First generation to college	64 (28%)	29 (29%)	47 (24%)	19 (21%)
Outside the ACTC site network	99 (44%)	51 (51%)	98 (49%)	45 (51%)
Highest degree obtained				
MD	37 (16%)	22 (22%)	60 (30%)	32 (36%)
PhD/EdD/PsyD/PharmD	86 (38%)	38 (38%)	134 (67%)	56 (63%)
MA/MS/MSN	59 (26%)	22 (22%)	4 (2.0%)	0 (0.0%)
BA/BS/BSN	43 (19%)	18 (18%)	1 (0.5%)	1 (1.1%)

^a^ One individual completed both tracks in separate years and is included twice in this table.

Abbreviation: ACTC, Alzheimer's Clinical Trial Consortium; IMPACT‐AD, Institute on Methods and Protocols for Advancement of Clinical Trials in ADRD.

**RESEARCH IN CONTEXT**

1. **Systematic review**: The authors used traditional tools to assess the literature. To our knowledge, relatively few training programs focus on clinical trials, fewer still on AD/ADRD.
2. **Interpretation**: We report five years of a successful training program focused on AD/ADRD clinical trials.
3. **Future directions**: Future efforts will continue to support previous trainees and enroll additional cohorts of interested investigators.

### 3.2 Evaluation of course components

**FIGURE 2 alz70441-fig-0002:**
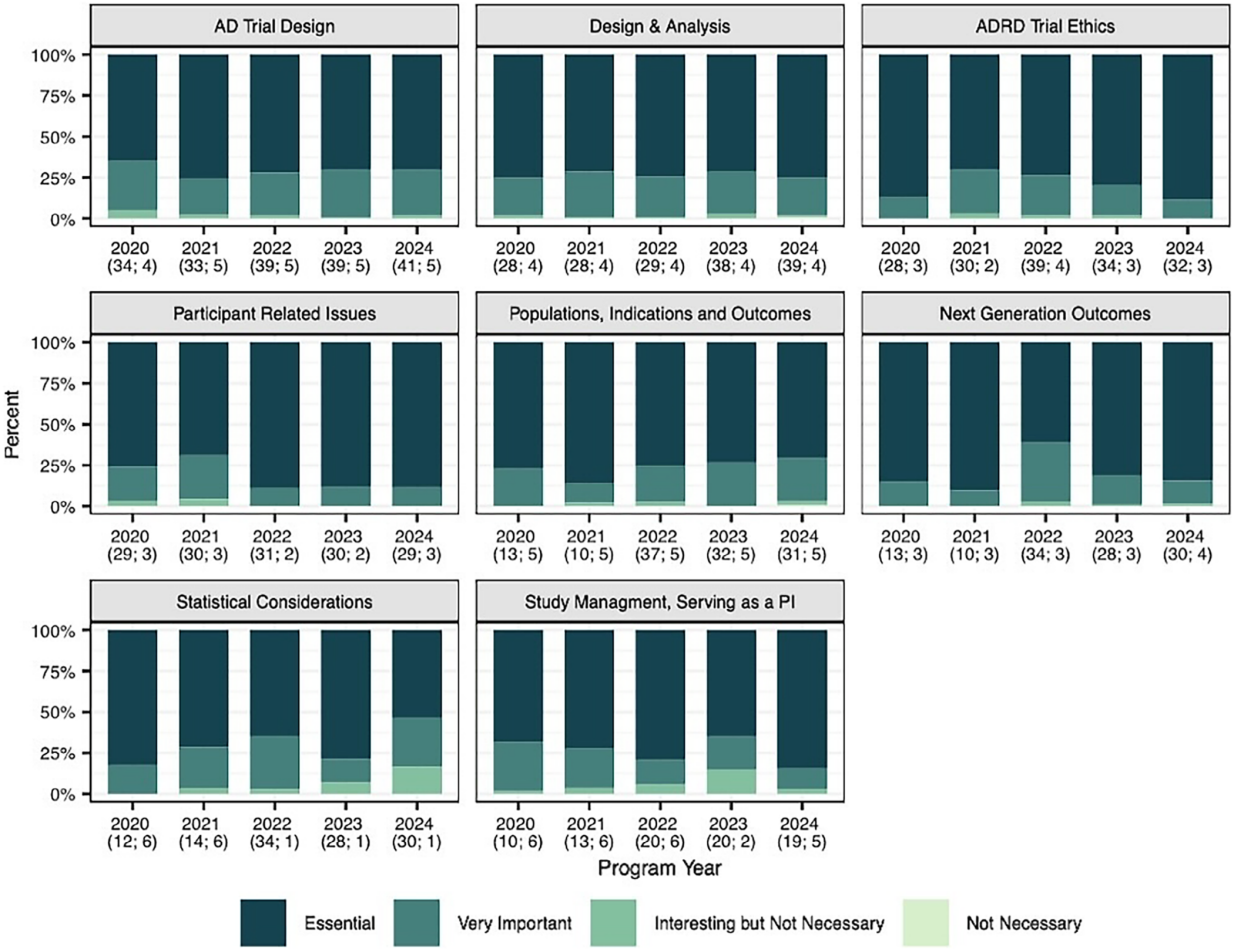
IMPACT‐AD session value ratings by year. Each panel presents trainee evaluation data across the years of IMPACT‐AD courses. Parentheses indicate (*N*, *n*), where *N* represents the number of participants that completed the volunteer survey and n represents the number of lectures comprising the session. AD, Alzheimer's disease; ADRD, Alzheimer's disease and related dementias; IMPACT‐AD, Institute on Methods and Protocols for Advancement of Clinical Trials in ADRD; PI, principal investigator.

IMPACT‐AD participants consistently viewed the core components of course content as “essential” or “very important,” as is illustrated in Figure 2. Across the years of course conduct, no element of the course received < 94% of participant ratings at these levels, with most rating each element each year as “essential.” Within these core components, modest changes occurred from year‐to‐year, including adjustments in specific elements of the didactic sessions and adjusted speakers. These adjustments appeared effective, as most sessions demonstrated consistently excellent evaluations or improvement over time.

### 3.3 Trainee knowledge assessments

Table 2 outlines trainee responses related to their pre‐ and post‐course knowledge. Among trial topic areas, trainees expressed the greatest need for education in design and analysis (64% indicated moderate or weak knowledge at baseline) and statistical considerations (75% indicated moderate or weak knowledge at baseline). They felt most knowledgeable in trial ethics and study management. Across topic areas, 48%, 39%, 10%, and 2% of trainees evaluated their change in knowledge based on the lectures as “very much increased,” “somewhat increased,” “slightly increased,” and “no change,” respectively. Among the core topics covered through the course, trainees expressed that they gained the most knowledge in the areas of participant‐related issues and populations, indications, and outcomes (Table 2).

**TABLE 2 alz70441-tbl-0002:** IMPACT‐AD didactic sessions evaluation summaries.

	Previous knowledge				Knowledge after lecture			
	Very strong (%)	Strong (%)	Moderate (%)	Weak (%)	Very much increased (%)	Somewhat increased (%)	Slightly increased (%)	No change (%)
AD trial design	19.3	26.9	38.5	15.3	48.9	41.1	8.2	1.8
Design & analysis	14.6	20.9	44.6	19.9	44.8	42.2	11.6	1.4
ADRD trial ethics	29.5	34.9	28.3	7.4	48.9	37.4	10.5	3.2
Participant‐related issues	16.4	35.4	41.0	7.2	56.0	31.5	10.8	1.7
Populations, indications, and outcomes	17.1	31.1	43.4	8.4	52.9	39.1	6.8	1.2
Next generation outcomes	14.5	24.0	45.3	16.2	51.9	37.6	7.7	2.7
Statistical considerations	11.1	13.3	53.8	21.8	40.5	44.3	12.1	3.2
Study management, serving as a PI	22.2	21.3	45.3	11.2	43.0	37.6	15.0	4.3
**Total**	**18.4**	**26.6**	**41.3**	**13.6**	**47.5**	**40.1**	**10.1**	**2.2**

Abbreviations: AD, Alzheimer's disease; ADRD, Alzheimer's disease and related dementias; IMPACT‐AD, Institute on Methods and Protocols for Advancement of Clinical Trials in ADRD; PI, principal investigator.

**FIGURE 3 alz70441-fig-0003:**
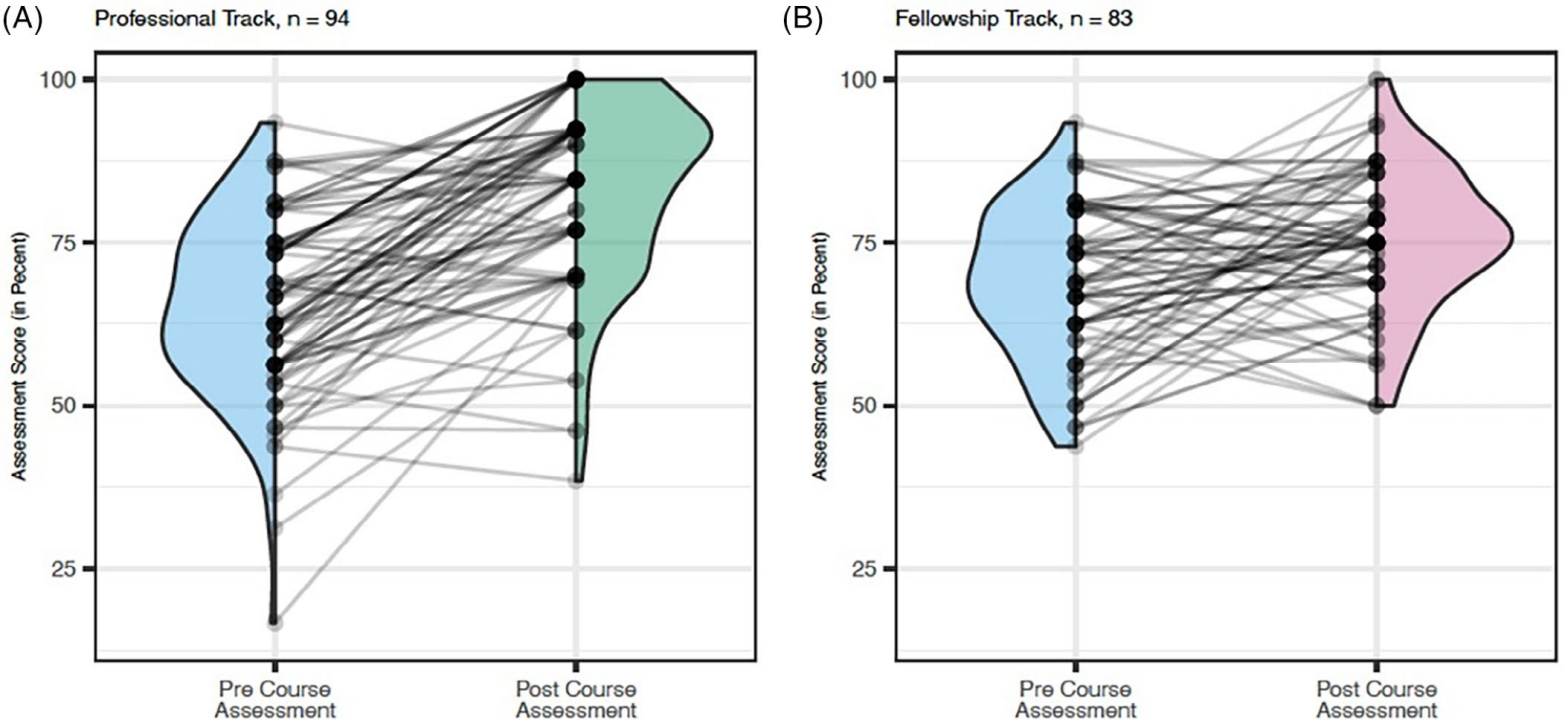
Pre‐course and post‐course assessment mean correct responses by program track. Participant level data are provided for each track, representing the objective testing outcomes for pre‐ and post‐course assessments. Violin plots illustrate the group level performances at the two time points and illustrate the overall increase after completion of the course in each track.

Each track demonstrated consistent increases in AD/ADRD trials knowledge, based on objective pre/post‐testing (Figure 3). The mean proportion of correct responses for the professional track increased from 65% to 83%. The fellowship track improved from 68% to 84% correct responses.

### 3.4 Long‐term outcomes

Among those completing annual[Fig alz70441-fig-0001], [Fig alz70441-fig-0002] follow‐up surveys (91/148, 61%),[Table alz70441-tbl-0002] > 84% remain in AD/ADRD trial careers, with slightly higher retention among the fellowship compared to the professional track (Table 3). Eighty percent have experienced career advances or milestones. Among this group, > 90% indicated that participating in IMPACT‐AD contributed to these career advances. For the fellowship track, 16 (33%) respondents had their trial protocol submitted and developed through the course funded, 15 (31%) had conducted their trial, and 5 (10%) had published their trial results. Among these, 88% to 100% indicated that IMPACT‐AD “somewhat” or “very much” contributed to this achievement.[Fig alz70441-fig-0003]

**TABLE 3 alz70441-tbl-0003:** Self‐reported career outcomes of IMPACT‐AD alumni–scholars (2020–2023).

	Professional track (*n* = 43)	Fellowship track (*n* = 48)
Still in AD/ADRD clinical trials	34 (79%)	43 (90%)
**Achievements**		
Career advancement/promotion	25 (48%)	25 (52%)
Research project funded	11 (26%)	22 (46%)
Acquired additional role/responsibilities	30 (70%)	36 (75%)
Impact of the course.		
IMPACT‐AD either “somewhat” or “very much” contributed to my career advancement/promotion.	25 (100%)	25 (100%)
IMPACT‐AD either “somewhat” or “very much” contributed to receiving funding for research project.	9 (82%)	21 (95%)

Abbreviations: AD, Alzheimer's disease; ADRD, Alzheimer's disease and related dementias; IMPACT‐AD, Institute on Methods and Protocols for Advancement of Clinical Trials in ADRD.

## 4 DISCUSSION

IMPACT‐AD has successfully completed five iterations of a cutting‐edge clinical trial training program that has been accessible to a broad pool of motivated professionals and scientists who were interested in expanding their knowledge and skills in AD/ADRD trials. Nearly 200 IMPACT‐AD alumni–scholars have undergone training, a relatively small but meaningful increase in the national workforce for these important studies. This, however, may represent an even more meaningful increase in the number of principal investigators who can lead trial sites and novel trials at their institutions. The course has also increased the number of individuals who can lead, manage, and conduct complex trials, as well as individuals with enhanced knowledge to serve as peer reviewers for scientific publications and research grant applications.[Table alz70441-tbl-0002], [Table alz70441-tbl-0003]

Though the main elements of course content have remained consistent since the inception of the course in 2020,^18^ there have been some key adjustments. After securing additional funding, the fellowship track was increased from 15 to 20 accepted trainees in 2022. Didactic presentations have been shortened to provide additional time for faculty panels and active discussion between trainees and faculty. Most importantly, in 2022, the course was held in person for the first time, realizing the original vision of an immersive experience for trainees to interact with each other and course faculty through not only didactic and workshop elements of a structured agenda but also through shared meals and activities.

The 5 years since launching IMPACT‐AD have also included remarkable progress in AD/ADRD research and treatment development. Since 2021, four new treatments have achieved US Food and Drug Administration approval (aducanumab, lecanemab, brexpiprazole, and donanemab), and the number and availability of new tools to support research, such as tau positron emission tomography imaging, plasma‐based biomarkers, and innovative biomarkers for non‐AD causes of neurodegeneration, are poised to further accelerate treatment development for all AD/ADRD conditions. We have accordingly made related adjustments to the course content. For example, new course debates have focused on whether lecanemab and donanemab have adequate data to support claims of disease modification,^24^ while ethics debates have included topics such as the role of placebo now that these treatments are clinically available.^25^

IMPACT‐AD participants expressed high levels of satisfaction with the course and its components. This satisfaction has been consistent from year to year, remarkably including among trainees who participated remotely compared to in‐person. We did observe modest increases in the ratings of several course components over time, which we attribute to minor adjustments made after each iteration of the program to increase its value. These adjustments included content updates but also subtle changes in approach, such as adjusted seating at the course to emphasize trainee participation.

Current leaders in AD/ADRD and other areas of trials scholarship do not adequately reflect the patient populations they aim to enroll in trials, that is, the greater disease suffering population.^26^ Cultivating a trials workforce that is representative of all demographic subgroups and professional backgrounds will be essential to achieving the goals of the national research enterprise, including infusing new ideas and improving the generalizability of trials through inclusive recruitment.^17^, ^27^ Ensuring accessibility to this novel educational resource has been a key success of the IMPACT‐AD program. The makeup of the alumni–scholars is notable for its inclusivity across a variety of demographic as well as professional backgrounds. As seen in Table 1, those accepted to the course are representative of the applicant pool, demonstrating that the strategy of broadly increasing awareness of the course as a career advancement opportunity was sufficient to ensure a diverse group of trainees.

IMPACT‐AD alumni–scholars have gone on to achieve career advances and routinely cite the course as playing a key role in their successes. We are aware of trainees being promoted within their organizations, being recruited to new positions at other organizations, and achieving key career milestones such as successful applications for grant funding. Importantly, these successes have been balanced across the two tracks, and nearly all indicated that participating in IMPACT‐AD contributed to this career growth. A notable 16 alumni–scholars from the fellowship track have had their trial protocol funded, 15 have conducted their trial, and 5 have already published their trial results.

Critically, among those completing longer term follow‐up surveys, > 84% of IMPACT‐AD alumni–scholars remain in AD/ADRD trial careers. Nevertheless, we recognize that these scholars continue to need support and mentorship. To achieve this, we recently initiated an annual Alumni–Scholar Conference, convening previous participants in person to enable continued support from IMPACT‐AD leadership and networking across cohorts. The latter is particularly important for cohorts that participated during COVID and for whom these meetings may represent the first in‐person interactions as part of the program. Though this element is pending grant funding to ensure its continued conduct, preliminary assessment of an initial pilot meeting indicated strong value to IMPACT‐AD alumni–scholars. Beyond this structured program, IMPACT‐AD leadership routinely holds “get together” convenings at meetings such as the Alzheimer's Association International Conference (AAIC) and the Clinical Trials in AD (CTAD) conference, and IMPACT‐AD alumni–scholars have even self‐organized such gatherings at other professional meetings. In partnership with the leadership of AAIC and CTAD, IMPACT‐AD alumni–scholars have had the opportunity to submit abstracts for consideration for early career symposia at these meetings. Collectively, the result of these efforts is a cohesive and connected network of investigators invested in the goals of the field and now with extensive connections to their trainee colleagues as well as IMPACT‐AD faculty to pursue meaningful scientific collaborations.

Though the course has been successful to date, it also has limitations. While the course has been broadly inclusive, trainees have been mostly neurologists, psychiatrists, neuroscientists, and trial staff members such as coordinators. Some groups, such as biostatisticians, regulatory specialists, and trial ethicists, have been less represented. We have also had relatively few trainees from industry. The course also takes a largely US‐centric approach, despite increasingly global approaches to drug development. Funded by the National Institute on Aging and the Alzheimer's Association, faculty and trainees in the main course are US‐based. We began a small pilot program with the Alzheimer's Association and the Global Brain Health Institute in 2022 to accept up to four fellowship track international trainees each year to address this need. Already this program has included trainees from seven non‐US countries. We have also begun to work with international partners to consider opportunities for similar courses in other global regions.

In conclusion, IMPACT‐AD is a formative program on AD/ADRD clinical trials that is occurring at a decisive juncture in the field when new treatments, biomarker advances, and better tools for AD/ADRD clinical trials have become available. The IMPACT‐AD alumni–scholar network, if appropriately supported and mentored, is poised to lead a new era of trials and progress in AD/ADRD research.

## CONFLICT OF INTEREST STATEMENT

Joshua D. Grill, PhD: research grants from NIA, Alzheimer's Association, BrightFocus Foundation, Lilly, Biogen, Genentech, and Eisai; personal compensation for editorial service to *Alzheimer's & Dementia*; travel paid for by the Alzheimer's Association. Margaret D. Mastrolorenzo: research support from the NIA and Alzheimer's Association. Heather Snyder, PhD: full‐time employee of the Alzheimer's Association. Maria Carillo, PhD: full‐time employee of the Alzheimer's Association. Ronald C. Petersen, MD, PhD: Roche, Inc.; Genentech, Inc.; Eli Lilly and Co., Eisai, Inc.; Novartis; and Novo Nordisk. Paul Aisen, MD, has research grants from NIH, the Alzheimer's Association, Lilly, and Eisai, and consults with Merck, Roche, Genentech, Abbvie, Biogen, ImmunoBrain Checkpoint, AltPep, Bristol Myers Squibb, and Neurimmune. Reisa Sperling, MD, has served as a paid consultant for AbbVie, AC Immune, Acumen, Alector, Apellis, Biohaven, Bristol Myers Squibb, Janssen, Oligomerix, Prothena, and Roche. She has received research funding from Eisai and Eli Lilly for public–private partnership clinical trials and receives research grant funding from the National Institute on Aging/National Institutes of Health, GHR Foundation, and the Alzheimer's Association. Her spouse, K. Johnson, reports consulting fees from Novartis, Merck, and Janssen. Kedir Hussen: research support from the NIA. Rema Raman, PhD: research support from the NIA, Alzheimer's Association, American Heart Association, and Eisai; travel paid for by the Alzheimer's Association. Author disclosures are available in the supporting information.

## CONSENT STATEMENT

This paper does not describe human subjects research and therefore there was no institutional review board approval needed.

## Supporting information



Supporting Information

## References

[1] Hebert LE , Weuve J , Scherr PA , Evans DA . Alzheimer disease in the United States (2010‐2050) estimated using the 2010 census. Neurology. 2013;80(19):1778‐1783. doi:10.1212/WNL.0b013e31828726f5 23390181 PMC3719424

[2] Brookmeyer R , Abdalla N , Kawas CH , Corrada MM . Forecasting the prevalence of preclinical and clinical Alzheimer's disease in the United States. Alzheimers Dement. 2018;14(2):121‐129. doi:10.1016/j.jalz.2017.10.009 29233480 PMC5803316

[3] National Plan To Address Alzheimer's Disease: 2017 Update. ASPE. 2017/09/08/T10:16:44‐04:00 2017.

[4] Bartels SJ , Naslund JA . The underside of the silver tsunami–older adults and mental health care. N Engl J Med. 2013;368(6):493‐496. doi:10.1056/NEJMp1211456 23343039

[5] Jeste DV . Geriatric psychiatry may be the mainstream psychiatry of the future. Am J Psychiatry. 2000;157(12):1912‐1914. doi:10.1176/appi.ajp.157.12.1912 11097950

[6] Institute of Medicine (US) Committee on the Future Health Care Workforce for Older Americans . Retooling for an aging America: building the health care workforce. National Academies Press (US); 2008. 25009893

[7] Warshaw GA , Bragg EJ . Preparing the health care workforce to care for adults with Alzheimer's disease and related dementias. Health Aff (Millwood). 2014;33(4):633‐641. doi:10.1377/hlthaff.2013.1232 24711325

[8] Freeman WD , Vatz KA , Griggs RC , Pedley T . The Workforce Task Force report: clinical implications for neurology. Neurology. 2013;81(5):479‐486. doi:10.1212/WNL.0b013e31829d8783 23783750 PMC3776536

[9] Sung NS , Crowley WF Jr , Genel M , et al. Central challenges facing the national clinical research enterprise. JAMA. 2003;289(10):1278‐1287. 12633190 10.1001/jama.289.10.1278

[10] Cummings J , Zhou Y , Lee G , Zhong K , Fonseca J , Cheng F . Alzheimer's disease drug development pipeline: 2024. Alzheimers Dement. 2024;10(2):e12465. doi:10.1002/trc2.12465 PMC11040692 38659717

[11] National Institute on Aging . NIA‐funded active Alzheimer's and related dementias clinical trials and studies. Accessed 2025, 2025. https://www.nia.nih.gov/research/ongoing‐AD‐trials

[12] Salloway SP , Sevingy J , Budur K , et al. Advancing combination therapy for Alzheimer's disease. 2020:e12073. doi:10.1002/trc2.12073 PMC7539671 33043108

[13] May M . Twenty‐five ways clinical trials have changed in the last 25 years. Nat Med. 2019;25(1):2‐5. doi:10.1038/s41591-018-0314-1 30617334

[14] Dorsey ER , Venuto C , Venkataraman V , Harris DA , Kieburtz K . Novel methods and technologies for 21st‐Century clinical trials: a review. JAMA Neurol. 2015;72(5):582‐588. doi:10.1001/jamaneurol.2014.4524 25730665 PMC4708881

[15] Gilliland CT , White J , Gee B , et al. The fundamental characteristics of a translational scientist. ACS Pharmacol Transl Sci. 2019;2:213‐216. 32259057 10.1021/acsptsci.9b00022 PMC7088880

[16] Bibbins‐Domingo K , Helman A , Dzau VJ . The imperative for diversity and inclusion in clinical trials and health research participation. JAMA. 2022;327(23):2283‐2284. doi:10.1001/jama.2022.9083 35579885

[17] Raman R , Aisen PS , Carillo MC , et al. Tackling a major deficiency of diversity in Alzheimer's disease therapeutic trials: an CTAD Task Force Report. J Prev Alzheimer's disease. 2022;9(3):388‐392. doi:10.14283/jpad.2022.50 35841239 PMC9098373

[18] Berkness T , Carrillo MC , Sperling R , et al. The Institute on Methods and Protocols for Advancement of Clinical Trials in ADRD (IMPACT‐AD): a novel clinical trials training program. J Prev Alzheimer's disease. 2021;8(3):286‐291. doi:10.14283/jpad.2021.12 34101785 PMC8019089

[19] Brich J , Jost M , Brustle P , Giesler M , Rijntjes M . Teaching neurology to medical students with a simplified version of team‐based learning. Neurology. 2017;89(6):616‐622. doi:10.1212/WNL.0000000000004211 28701497

[20] Freeman S , Eddy SL , McDonough M , et al. Active learning increases student performance in science, engineering, and mathematics. Proc Nat Acad Sci USA. 2014;111(23):8410‐8415. doi:10.1073/pnas.1319030111 24821756 PMC4060654

[21] Granero Lucchetti AL , Ezequiel ODS , Oliveira IN , Moreira‐Almeida A , Lucchetti G . Using traditional or flipped classrooms to teach “Geriatrics and Gerontology”? Investigating the impact of active learning on medical students' competences. Med Teach. 2018;40(12):1248‐1256. doi:10.1080/0142159X.2018.1426837 29355063

[22] Straus SE , Sackett DL . Clinician‐trialist rounds: 7. Mentoring: why every clinician‐trialist needs to get mentored. Clin Trials. 2011;8(6):765‐767. doi:10.1177/1740774511423948 22167113

[23] Sambunjak D , Straus SE , Marusic A . Mentoring in academic medicine: a systematic review. JAMA. 2006;296(9):1103‐1115. doi:10.1001/jama.296.9.1103 16954490

[24] Planche V , Villain N . Advocating for demonstration of disease modification‐have we been approaching clinical trials in early Alzheimer disease incorrectly?. JAMA Neurol. 2023;80(7):659‐660. doi:10.1001/jamaneurol.2023.0815 37093582

[25] Grill JD , Karlawish J . Implications of FDA approval of a first disease‐modifying therapy for a neurodegenerative disease on the design of subsequent clinical trials. Neurology. 2021;97(10):496‐500. doi:10.1212/WNL.0000000000012329 34088880 PMC8448555

[26] Getz K , Faden L . Racial disparities among clinical research investigators. Am J Ther. 2008;15(1):3‐11. doi:10.1097/MJT.0b013e31815fa75a 18223347

[27] Grill JD , Sperling RA , Raman R . What should the goals be for diverse recruitment in Alzheimer clinical trials?. JAMA Neurol. 2022;79(11):1097‐1098. doi:10.1001/jamaneurol.2022.2274 35969392

